# Revealing potential diagnostic gene biomarkers of septic shock based on machine learning analysis

**DOI:** 10.1186/s12879-022-07056-4

**Published:** 2022-01-19

**Authors:** Yonghua Fan, Qiufeng Han, Jinfeng Li, Gaige Ye, Xianjing Zhang, Tengxiao Xu, Huaqing Li

**Affiliations:** 1 Emergency Medicine Department, The Second Affiliated Hospital of Shandong First Medical University, 366 Taishan Street, Tai’an, 271000 Shandong China; 2 ICU, The Second Affiliated Hospital of Shandong First Medical University, 366 Taishan Street, Tai’an, 271000 Shandong China

**Keywords:** DEmRNAs (differentially expressed mRNAs), Diagnostic gene biomarkers, Machine learning analysis, Prognostic, Septic shock

## Abstract

**Background:**

Sepsis is an inflammatory response caused by infection with pathogenic microorganisms. The body shock caused by it is called septic shock. In view of this, we aimed to identify potential diagnostic gene biomarkers of the disease.

**Material and methods:**

Firstly, mRNAs expression data sets of septic shock were retrieved and downloaded from the GEO (Gene Expression Omnibus) database for differential expression analysis. Functional enrichment analysis was then used to identify the biological function of DEmRNAs (differentially expressed mRNAs). Machine learning analysis was used to determine the diagnostic gene biomarkers for septic shock. Thirdly, RT-PCR (real-time polymerase chain reaction) verification was performed. Lastly, GSE65682 data set was utilized to further perform diagnostic and prognostic analysis of identified superlative diagnostic gene biomarkers.

**Results:**

A total of 843 DEmRNAs, including 458 up-regulated and 385 down-regulated DEmRNAs were obtained in septic shock. 15 superlative diagnostic gene biomarkers (such as RAB13, KIF1B, CLEC5A, FCER1A, CACNA2D3, DUSP3, HMGN3, MGST1 and ARHGEF18) for septic shock were identified by machine learning analysis. RF (random forests), SVM (support vector machine) and DT (decision tree) models were used to construct classification models. The accuracy of the DT, SVM and RF models were very high. Interestingly, the RF model had the highest accuracy. It is worth mentioning that ARHGEF18 and FCER1A were related to survival. CACNA2D3 and DUSP3 participated in MAPK signaling pathway to regulate septic shock.

**Conclusion:**

Identified diagnostic gene biomarkers may be helpful in the diagnosis and therapy of patients with septic shock.

**Supplementary Information:**

The online version contains supplementary material available at 10.1186/s12879-022-07056-4.

## Background

Sepsis is an inflammatory response caused by infection with pathogenic microorganisms. The body shock caused by it is called septic shock. Sepsis is a reaction to systemic infections [1, 2]. Septic shock, associated with critical hypotension, is common acute diseases in the ICU (intensive care unit) [2, 3]. It is estimated that about 8 million people worldwide die from sepsis (usually septic shock) every year, and abnormalities in the circulatory system, cells, and metabolism can significantly increase mortality [1, 4].

Most of septic shock is caused by microbial infections (bacteria, viruses, fungi, etc.) [5]. In early microbial infections, humoral reactions are activated, in which immune cells (macrophages, neutrophils, etc.) recognize and destroy invading organisms [6]. Reduced blood vessel volume, cardiac dysfunction and peripheral vasodilation are major causes of septic shock [6, 7]. In view of this, active fluid resuscitation and anti-infective symptomatic treatment are performed in these patients [8–10]. However, 28-day and hospital mortality in patients remain very high [8]. Moreover, the probability of re-admission after discharge from hospital is higher than that of ordinary ICU patients, and a considerable proportion of patients have cognitive dysfunction after treatment [11–13].

Diagnosis and prognostic detection of diseases at the molecular level are now the general trend of development, which is also widely used by researchers in sepsis [14, 15]. Mohammed et al. used high-throughput sequencing technology to identify potential biomarkers and signaling pathways related to septic shock [16]. In addition, some researchers use TSD (transcriptomic signature distance) and meta-analysis to analyze the transcriptome data of septic shock patients [17, 18]. Machine learning is a branch of computer science and statistics that play an important role in the detection, diagnosis and treatment of diseases [19, 20]. Machine learning has also been used to study septic shock [21, 22]. However, most of these studies use machine learning to predict the progression of septic shock. Machine learning is rarely used to identify potential diagnostic and prognostic biomarkers of septic shock. Therefore, in order to identify potential diagnostic gene biomarkers of septic shock, machine learning method was performed, followed by prognostic analysis in this study. Our study could be valuable in understanding the pathological mechanism of septic shock and exploring novel diagnostic gene biomarker for the diagnostic and therapy of the disease.

## Methods

### Database

GEO [23] (Gene Expression Omnibus) database, mainly based on chip data, is developed by NCBI (National Center for Biotechnology Information). GSE4607, GSE13904, GSE26378, GSE26440, GSE65682 and GSE95233 data sets were obtained (Table 1). The original file was downloaded and the RMA algorithm was used for background adjustment and normalization. If multiple probes correspond to the same gene, the average value was taken. Among them, GSE4607, GSE13904, GSE26378, GSE26440 data sets were used for differential expression analysis and machine learning (test set), and GSE65682 data set was used for survival analysis. The GSE95233 data set was used for electronic expression verification of gene biomarkers (validation set). In this study, the GSE65682 data set was based on the chip data of GPL 13667 platform, and GSE4607, GSE13904, GSE26378, GSE26440 and GSE95233 data sets were based on the GPL570 platform. In order to avoid the difference caused by the detection technology of different platforms, the GSE65682 data set was not analyzed together with other data sets. Since the GSE4607, GSE13904, GSE26378 and GSE26440 data sets all came from GPL570 platform. Batch effect processing using the SVA package showed that the results of batch effect between the four data sets was not significant (Additional file 1: Fig. S1).

**Table 1:** Dataset retrieved from the GEO database

GEO ID	Samples (Normal control:Septic shock)	Type	Platform	Year	Author	Type
GSE4607	15:69	Blood	GPL 570	2006	Hector R Wong	mRNA
GSE13904	18:106	Blood	GPL 570	2008	Hector R Wong	mRNA
GSE26378	21:82	Blood	GPL 570	2011	Wong HR	mRNA
GSE26440	32:98	Blood	GPL 570	2011	Wong HR	mRNA
GSE65682	42:479	Blood	GPL 13667	2015	Scicluna BP	mRNA
GSE95233	22:102	Blood	GPL 570	2017	Pachot A	mRNA

### Identification of DEmRNAs (differentially expressed mRNAs)

In this study, Limma and metaMA packages were executed for identification of the DEmRNAs. The inverse normal method was used in the metaMA software package to merge P values. The FDR (false discovery rate) is the result obtained by repeating the test and correction of the original P value by the Benjamin and Hochberg methods [24, 25]. The FDR < 0.01 and |Combined.ES (effect size)|> 1.5 were screening thresholds of DEmRNAs.

### Functional enrichment

To identify the function of identified genes, the DAVID (Database for Annotation, Visualization and Integrated Discovery, https://david.ncifcrf.gov/) database was used for GO (Gene Ontology, http://www.geneontology.org/) and KEGG (Kyoto encyclopedia of genes and genomes, http://www.genome.jp/kegg/pathway.html) functional enrichment analysis [26–28]. P < 0.05 was the threshold of significantly enriched GO and KEGG terms.

### Identification of the superlative diagnostic gene biomarkers

Firstly, the R language in glmnet package was used to reduce data dimensions. The package not only has a large number of models, but also is much faster [29]. Secondly, the random forest algorithm was used to sort the importance of mRNA according to the Mean Decrease Accuracy value from large small. Then, the superlative number of features was identified by adding one differentially expressed mRNA at a time in a top down forward-wrapper approach. The superlative DEmRNAs with diagnostic value was selected for septic shock to establish a classification model including DT (decision tree), SVM (support vector machine) and RF (random forests). The ‘rpart’ packet in R (https://cran.r-project.org/web/packages/rpart/), ‘e1071’ package in R (https://cran.r-project.org/web/packages/e1071/index.html) and ‘random forests’ packet (https://cran.r-project.org/web/packages/randomForest/) was used to establish the DT model, SVM model and RF model, respectively. Tenfold cross-validation was used to compare the average misjudgment rates of the three models. Tenfold cross-validation was used to avoid the overfitting effect [30, 31]. The diagnostic ability of classification prediction was evaluated by the accuracy, sensitivity, specificity, and AUC (area under curve) values in the ROC (receiver operating characteristic) curve. Subsequently, the Matthew’s Correlation Coefficient of the model was calculated using the mcc function in the mltools package (https://pypi.org/project/mltools/1.0.2/).

### Electronic expression verification, diagnostic and prognostic analysis of superlative diagnostic gene biomarkers

The GSE95233 data set (124 blood samples from 102 cases and 22 normal controls) was used for electronic expression verification. The GSE65682 data set (521 blood samples from 479 cases and 42 normal controls) contains 28 days of survival information of patients. This data set was used to further analyze the diagnostic and survival ability of key diagnostic gene biomarkers.

### In vitro validation of identified DEmRNAs

The inclusion criteria for patients were diagnosed with septic shock. Detailed inclusion criteria for patients with septic shock were as follows: (1) the body temperature > 38 ℃ or < 36 ℃; (2) heart rate > 90 times per minute or greater than 2 standard deviations in the normal heart rate range of different ages; (3) respiratory rate > 20 times per minute or PaCO2 (partial pressure of carbon dioxide in artery) < 32 mmHg; (4) white blood cell count > 12.0 × 10^9^/L or < 4.0 × 10^9^/L, or more than 10% immature neutrophils; (5) patients with initial septic shock; (6) patients had cardiovascular organ dysfunction, acute respiratory distress syndrome, dysfunction of two or more other organs; (7) patients had complete clinical data, including gender, age, height, weight, etc. Patients with a history of cancer or other diseases, chemotherapy, radiotherapy, etc., and incomplete clinical data were excluded. The individuals in the normal control group were gender and age matched with the case group and had no disease before and within 2 weeks after sampling. Those individual who took glucocorticoids, had a history of febrile disease or any chronic/acute disease that is slightly associated with inflammation within 2 weeks of sampling were excluded.

According to the above criteria for septic shock, 16 blood samples from 8 patients and 8 normal controls were obtained for RT-PCR (real-time polymerase chain reaction). Total RNA was extracted by using RNAliquid ultra-speed whole blood (liquid sample) kit (RN2602, Beijing Huitian Oriental Technology Co., Ltd.). FastQuant cDNA synthesis kit (KR106, TIANGEN) was used to synthesize the cDNA. RT-PCR was performed using SuperReal PreMix Plus (SYBR Green) SuperReal reagent (FP205, TIANGEN). Each experiment was repeated three times. GAPDH (glyceraldehyde-3-phosphate dehydrogenase) and ACTB (actin beta) were used as internal control for gene detection. The relative expression levels were calculated as fold-changes using the 2^−ΔΔCt^ method [32].

This study was approved by the ethics committee the Second Affiliated Hospital of Shandong First Medical University (20200406).

### Statistical analysis

The GraphPad Prism was used to perform all statistical analyses. The significance cutoff of RT-PCR was P = 0.05 (Duncan’s multiple range test). One-way ANOVA (analysis of variance) with orthogonal contrasts and mean comparison procedures were used to detect differences between cases and normal controls. Experiments were independently repeated at least 3 times.

## Results

### DEmRNAs

**Fig. 1:**
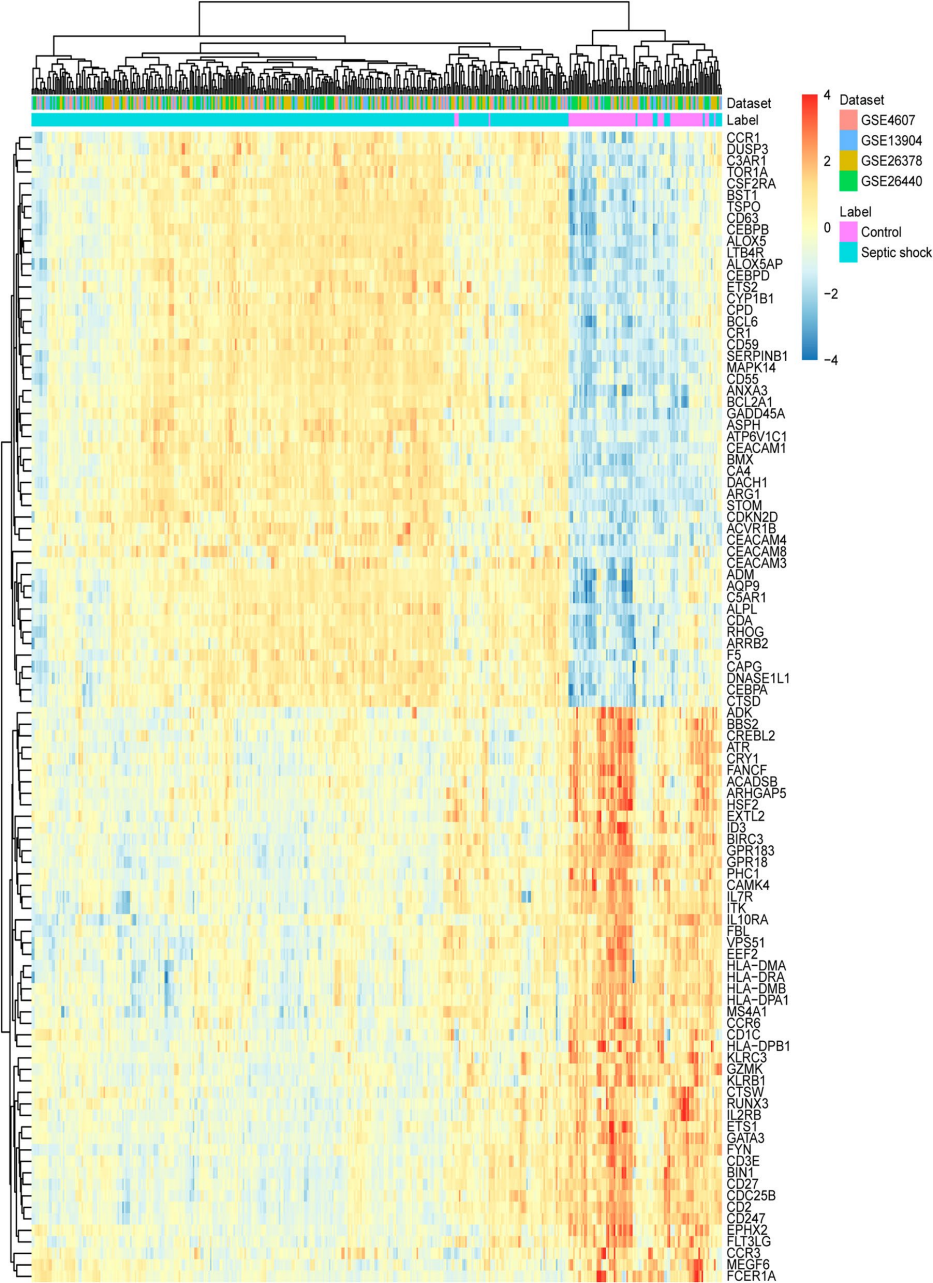
Heat map of top 100 DEmRNAs. The figure shows the bidirectional hierarchical clustering results of the top 100 DEmRNAs and samples. A full chain method combined with Euclidean distance is used to establish clustering (row: DEmRNA, column: sample). The color cluster tree on the right indicates the relative expression level of mRNA. Red indicates below the reference channel. Blue indicates the above reference

According to screening criteria of FDR < 0.01 and |Combined.ES|> 1.5, a total of 843 DEmRNAs were identified. Among which, 458 were up-regulated and 385 were down-regulated (Additional file 3: Table S1). The heat map of top 100 DEmRNAs is shown in the Fig. 1.

### Functional enrichment analysis of DEmRNAs

**Fig. 2:**
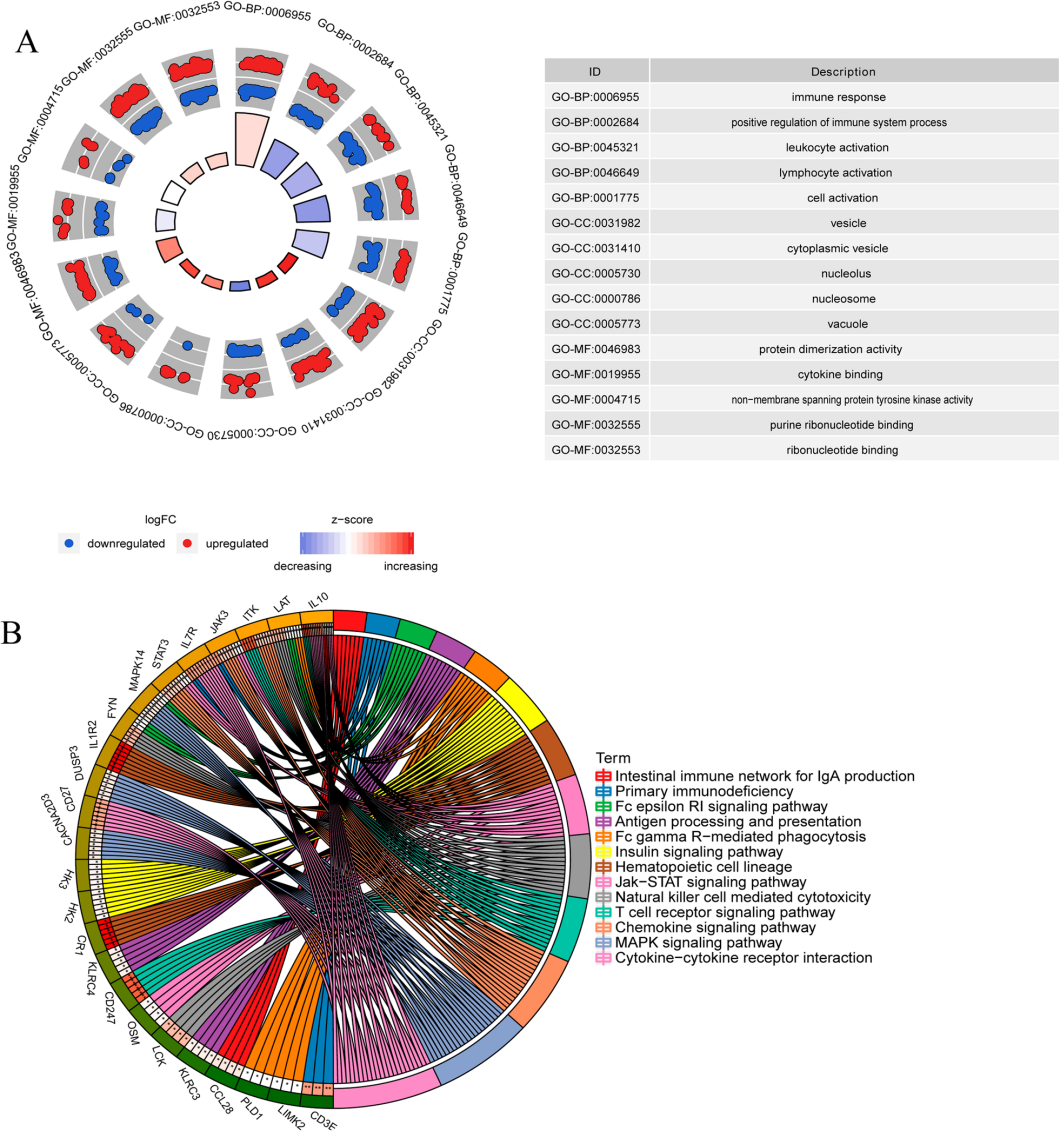
Top 15 significantly enriched GO and top 13 significantly enriched KEGG terms of all DEmRNAs. **A** Top 15 significantly enriched GO terms enrichment of DEmRNAs. The z-score clustering in the GO terms of all DEmRNAs is shown below. Red represents mRNA up-regulation and blue represents mRNA down-regulation. *GO* Gene Ontology, *BP* biological process, *CC* cell composition, *MF* molecular function. **B** Top 13 significantly enriched KEGG terms of DEmRNAs. The KEGG different colors represent different signaling pathways [26–28]. *KEGG* Kyoto encyclopedia of genes and genomes

In order to understand the potential biological function of DEmRNAs, GO and KEGG functional enrichment analysis were performed. In GO terms of BP (biological process), all DEmRNAs were mainly involved in immune response, positive regulation of immune system process and leukocyte activation. In GO terms of CC (cell composition), all DEmRNAs were mainly involved in vesicle, cytoplasmic vesicle and nucleolus. In GO terms of MF (molecular function), all DEmRNAs were mainly involved in protein dimerization activity, cytokine binding and non-membrane spanning protein tyrosine kinase activity. The result is shown in Fig. 2A. Several signaling pathways in the KEGG enrichment analysis were identified, such as T cell receptor signaling pathway, primary immunodeficiency, MAPK signaling pathway, Jak–STAT signaling pathway and Fc epsilon RI signaling pathway (Fig. 2B). Among the 15 superlative diagnostic gene biomarkers, CACNA2D3 and DUSP3 participated in the MAPK signaling pathway.

### Identification of superlative diagnostic gene biomarkers

**Fig. 3:**
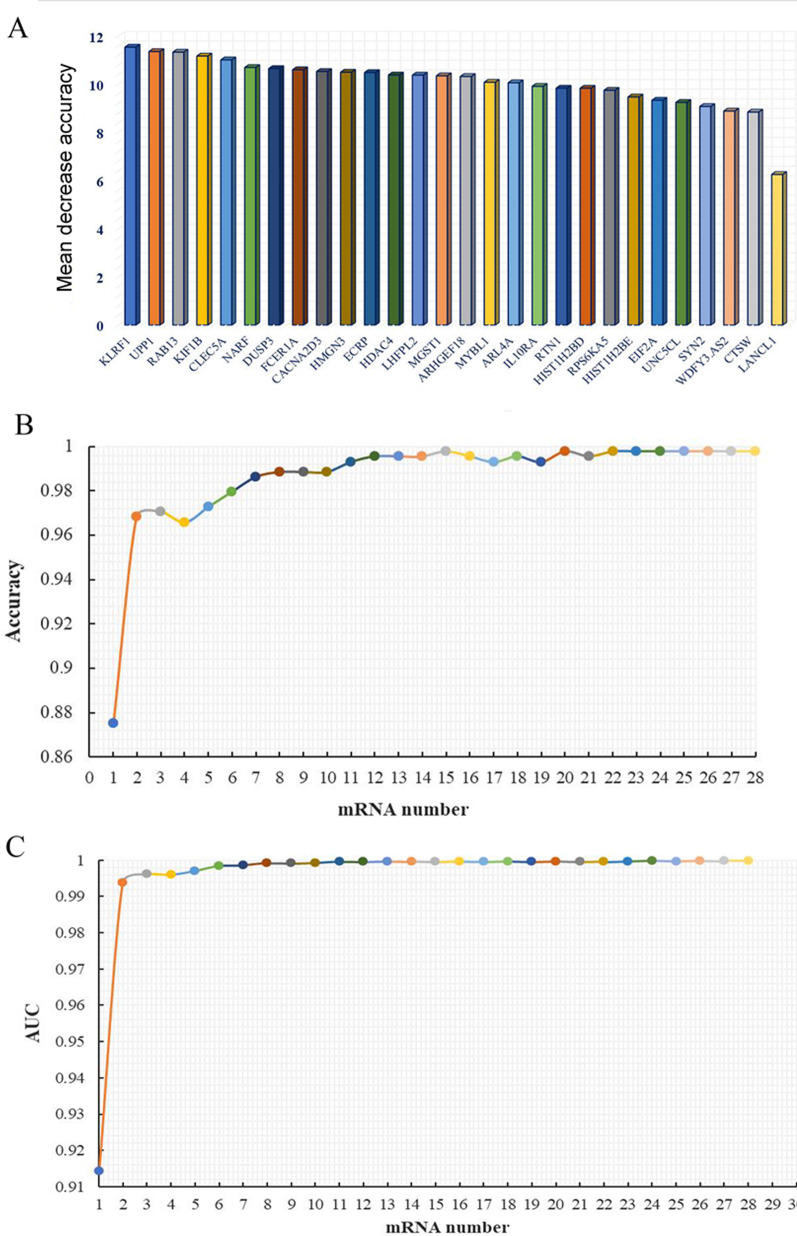
The importance ranking of 28 DEmRNAs and the trend graph of accuracy rate and AUC increasing with the number of mRNAs. **A** Importance ranking of 28 DEmRNAs; **B** Trend graph of accuracy rate increasing with the number of mRNAs; **C** Trend graph of AUC increasing with the number of mRNAs. *AUC* area under curve

**Fig. 4:**
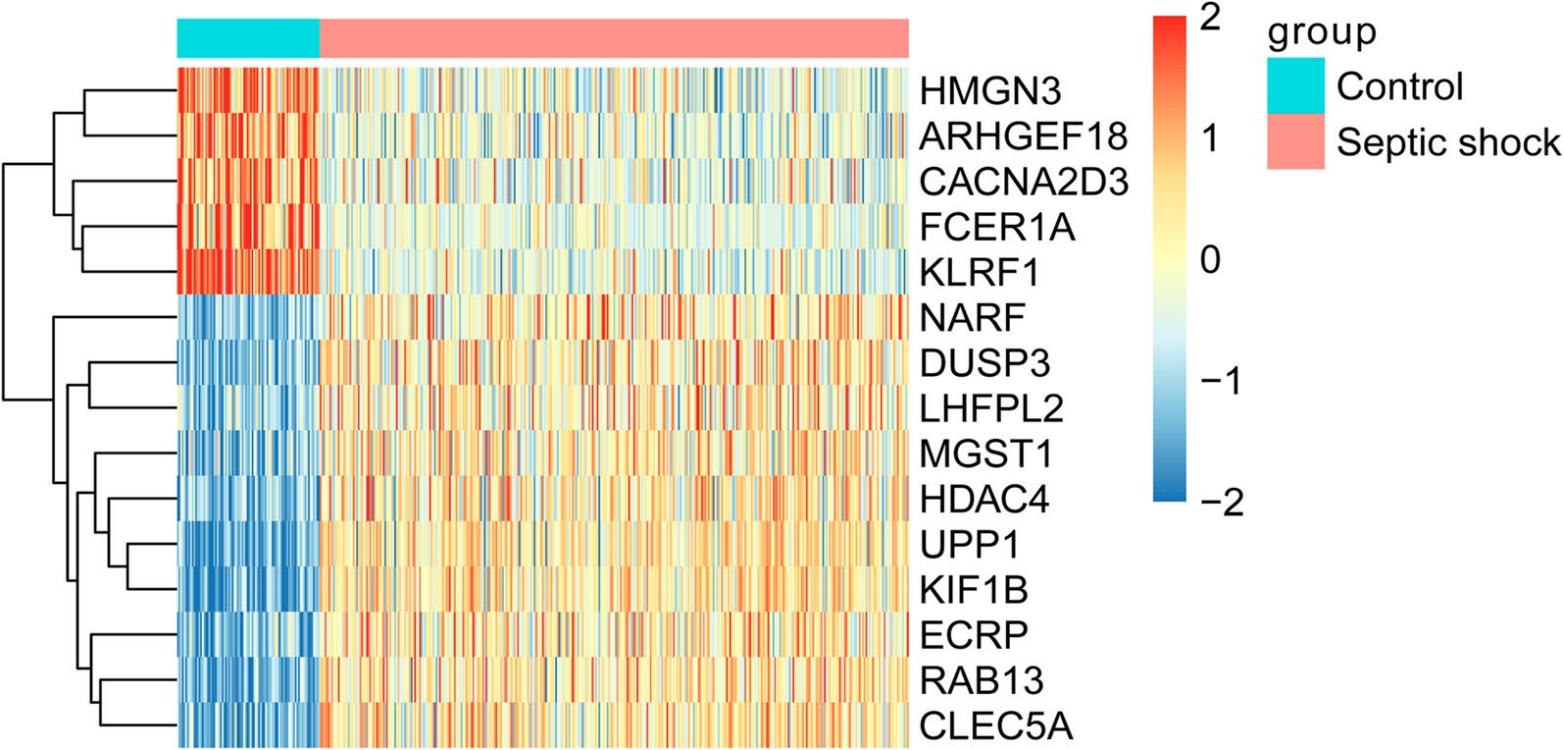
Heat map of 15 superlative diagnostic gene biomarkers. A full chain method combined with Euclidean distance is used to establish clustering. Each row represents a diagnostic biomarker, and each column represents a sample. The color cluster tree on the right indicates the relative expression level of mRNA. Red indicates below the reference channel. Blue indicates the above reference

After reducing data dimensions, a total of 28 DEmRNAs were retained (Table 2). 28 DEmRNAs were ranked in order of importance according to Mean Decrease Accuracy value (Fig. 3A). According to the sequence of RF sequencing results, one mRNA was added successively from top to bottom. The RF algorithm was used for classification. The tenfold cross-validation was used to obtain the accuracy rate and AUC (Fig. 3B, C). It can be seen that when the number of mRNAs reached 15, the accuracy reached the maximum value for the first time. Therefore, the first 15 DEmRNAs (KLRF1, UPP1, RAB13, KIF1B, CLEC5A, NARF, DUSP3, FCER1A, CACNA2D3, HMGN3, ECRP, HDAC4, LHFPL2, MGST1 and ARHGEF18) were selected as the superlative diagnostic gene biomarkers. The heat map analysis of the 15 superlative diagnostic gene biomarkers is shown in Fig. 4.

**Table 2:** 28 differentially expressed mRNAs after reducing data dimensions

ID	Symbol	Combined. effect size	P value	False discovery rate	Up/Down
1845	DUSP3	1.966918	< 2.22e−16	< 1.64e−15	Up
3017	HIST1H2BD	1.805068	< 2.22e−16	< 1.64e−15	Up
4257	MGST1	1.871323	< 2.22e−16	< 1.64e−15	Up
5872	RAB13	2.507966	< 2.22e−16	< 1.64e−15	Up
6854	SYN2	1.562134	< 2.22e−16	< 1.64e−15	Up
7378	UPP1	2.807998	< 2.22e−16	< 1.64e−15	Up
8344	HIST1H2BE	1.705221	< 2.22e−16	< 1.64e−15	Up
9759	HDAC4	1.892591	< 2.22e−16	< 1.64e−15	Up
10124	ARL4A	1.554429	< 2.22e−16	< 1.64e−15	Up
10184	LHFPL2	1.50707	< 2.22e−16	< 1.64e−15	Up
23095	KIF1B	2.805012	< 2.22e−16	< 1.64e−15	Up
23601	CLEC5A	2.375364	< 2.22e−16	< 1.64e−15	Up
26502	NARF	1.747345	< 2.22e−16	< 1.64e−15	Up
643332	ECRP	1.947246	< 2.22e−16	< 1.64e−15	Up
404201	WDFY3-AS2	1.551524	< 2.22e−16	< 1.64e−15	Up
1521	CTSW	− 1.56967	< 2.22e−16	< 1.64e−15	Down
2205	FCER1A	− 2.07841	< 2.22e−16	< 1.64e−15	Down
3587	IL10RA	− 1.56804	< 2.22e−16	< 1.64e−15	Down
4603	MYBL1	− 1.74565	< 2.22e−16	< 1.64e−15	Down
6252	RTN1	− 1.74447	< 2.22e−16	< 1.64e−15	Down
9252	RPS6KA5	− 1.70288	< 2.22e−16	< 1.64e−15	Down
9324	HMGN3	− 1.88269	< 2.22e−16	< 1.64e−15	Down
10314	LANCL1	− 1.56694	< 2.22e−16	< 1.64e−15	Down
23370	ARHGEF18	− 1.78719	< 2.22e−16	< 1.64e−15	Down
51348	KLRF1	− 2.74432	< 2.22e−16	< 1.64e−15	Down
55799	CACNA2D3	− 1.58142	< 2.22e−16	< 1.64e−15	Down
83939	EIF2A	− 1.72077	< 2.22e−16	< 1.64e−15	Down
222643	UNC5CL	− 1.51897	< 2.22e−16	< 1.64e−15	Down

**Fig. 5:**
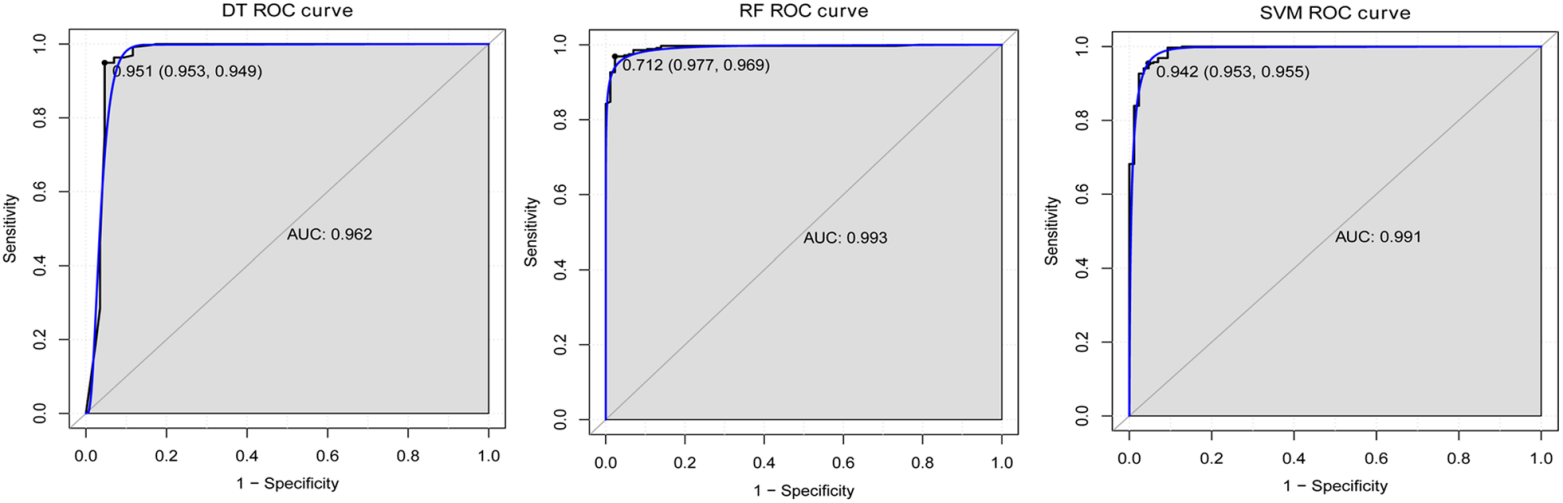
ROC curve of DT, RF and SVM classifier. *AUC* area under curve, *ROC* receiver operating characteristic

**Fig. 6:**
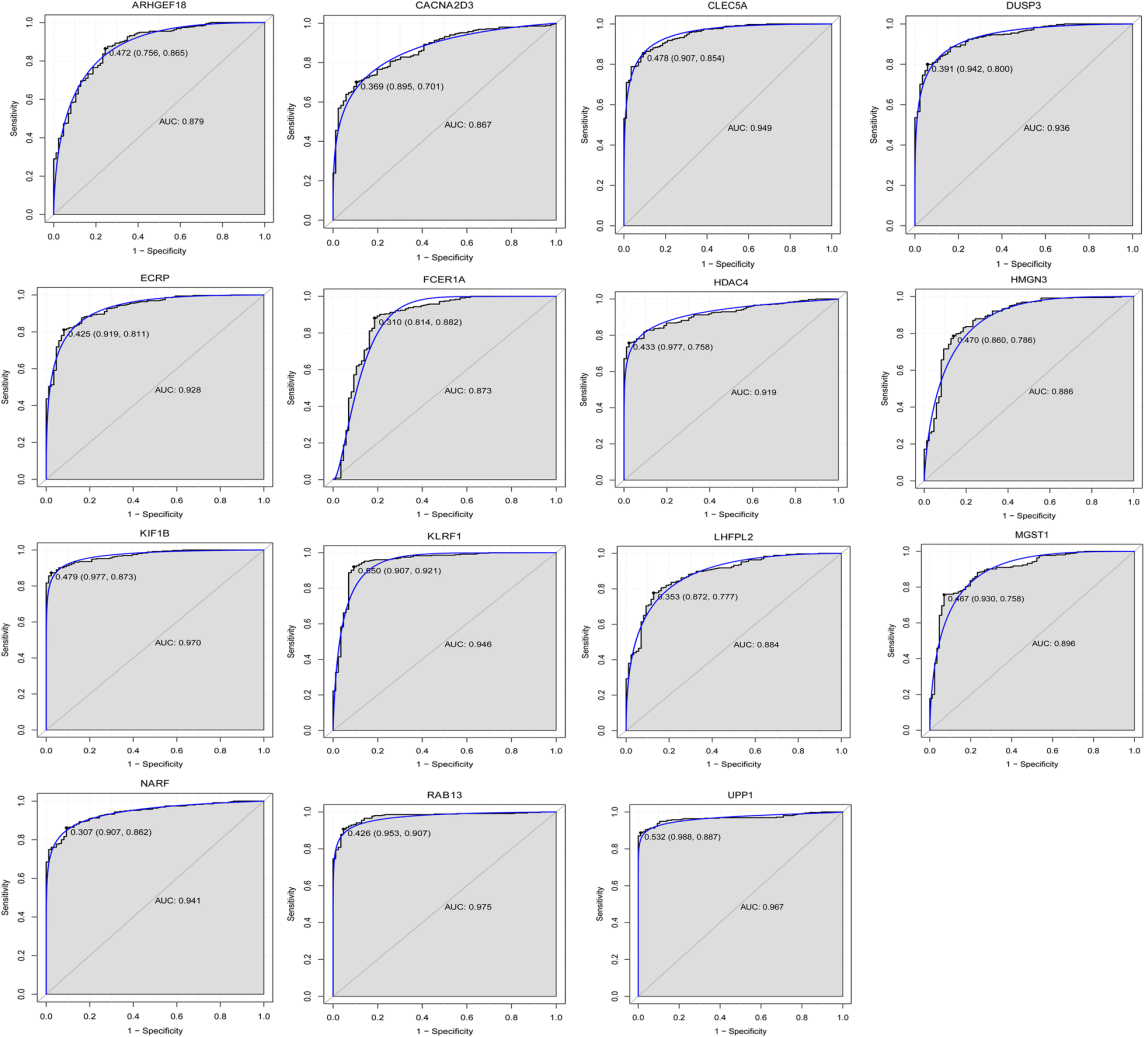
ROC curve of 15 superlative diagnostic gene biomarkers. *AUC* area under curve, *ROC* receiver operating characteristic

Classification models were constructed based on the screened 15 genes. The RF model had the highest accuracy. The accuracy, sensitivity, specificity and AUC of each model using the tenfold cross-validation process is listed in Table 3. In addition, the AUC in the ROC curve of DT, RF and SVM, was respectively 0.962, 0.993, and 0.991 (Fig. 5). The diagnostic efficacy of the model composed of these 15 genes was also validated using the GSE95233 data set. The results showed that in the validation set, our diagnostic model also showed better performance (Additional file 2: Fig. S2B–D). In addition, the Matthew’s Correlation Coefficient also showed that our model showed high accuracy in the test set. Although the performance in the verification set was not as good as the test set, it also had better accuracy (Additional file 4: Table S2). Significantly, of 15 superlative diagnostic gene biomarkers, the AUC values of CLEC5A, DUSP3, ECRP, HDAC4, KIF1B, KLRF1, NARF, RAB13 and UPP1 were higher than 0.9, the sensitivity and specificity were higher than 0.8 in the ROC curve analysis (Fig. 6).

**Table 3:** Ten-fold cross-validation results of each model

Classifier	Accuracy	Sensitivity	Specificity	AUC
DT	0.935	0.949	0.953	0.962
RF	0.978	0.969	0.977	0.993
SVM	0.963	0.955	0.953	0.991

*DT* decision tree, *RF* random forest, *SVM* support vector machines, *AUC* area under curve

### Electronic expression verification, diagnosis and prognostic analysis of superlative diagnostic gene biomarkers

**Fig. 7:**
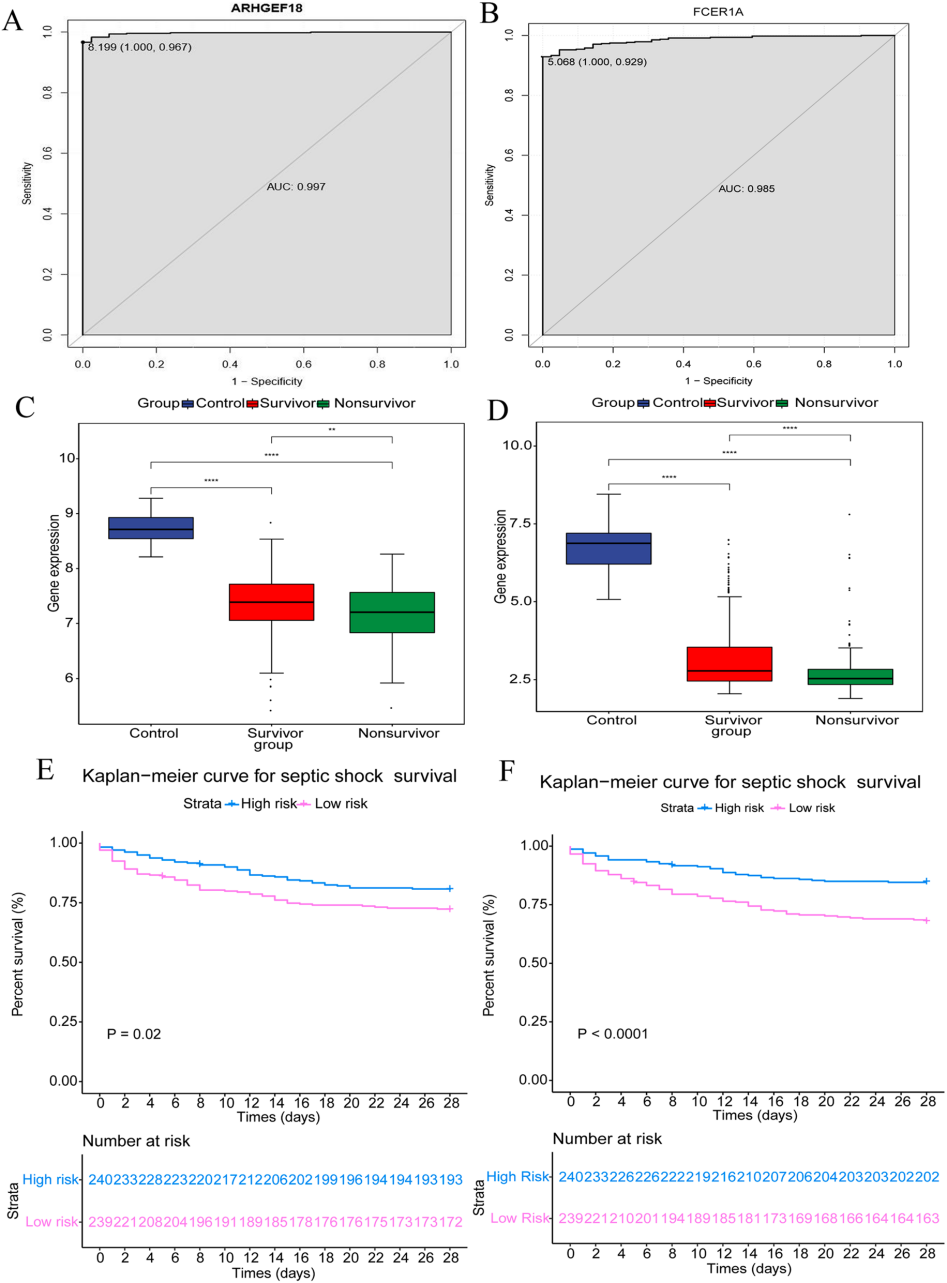
Diagnosis and prognostic analysis of ARHGEF18 and FCER1A in GSE65682 data sets. **A** ROC curve of ARHGEF18; **B** ROC curve of FCER1A; **C** Box plot of ARHGEF18; **D** Box plot of FCER1A; **E** Survival curve of ARHGEF18; **F** Survival curve of FCER1A. *AUC* area under curve, *ROC* receiver operating characteristic

In order to further verify the expression of 15 diagnostic gene biomarkers, expression verification was performed using the GSE95233 data set. The results showed that ARHGEF18, CACNA2D3, FCER1A, HMGN3 and KLRF1 were significantly down-regulated in disease group, while CLEC5A, DUSP3, ECRP, HDAC4, KIF1B, LHFPL2, MGST1, NARF, RAB13 and UPP1 were significantly down-regulated compared with normal control group (Additional file 2: Fig. S2A). This verification result was completely consistent with the previous analysis result. The data set of GSE65682 was selected to perform further diagnosis and prognostic analysis of identified superlative diagnostic gene biomarkers (Fig. 7). The analysis results showed that only ARHGEF18 and FCER1A were related to survival. The AUC, sensitivity and specificity of ARHGEF18 were respectively 0.997, 0.967 and 1.000. The AUC, sensitivity and specificity of FCER1A were 0.985, 0.929 and 1.000, respectively (Fig. 7A, B). Box plots showed the expression levels of ARHGEF18 and FCER1A in different populations (Fig. 7C, D). In the survivor population, the expression levels of ARHGEF18 and FCER1A were significantly down-regulated, which was consisted with the bioinformatics analysis. The level of gene expression was the lowest among dead people. ARHGEF18 and FCER1A may influence the treatment effect of patients to a certain extent. Then the online survival software package (https://cran.r-project.org/web/packages/survival/index.html) was used to analyze the prognostic value of ARHGEF18 and FCER1A. The results showed that ARHGEF18 and FCER1A were significantly negatively correlated with survival (Fig. 7E, F).

### RT-PCR validation

**Fig. 8:**
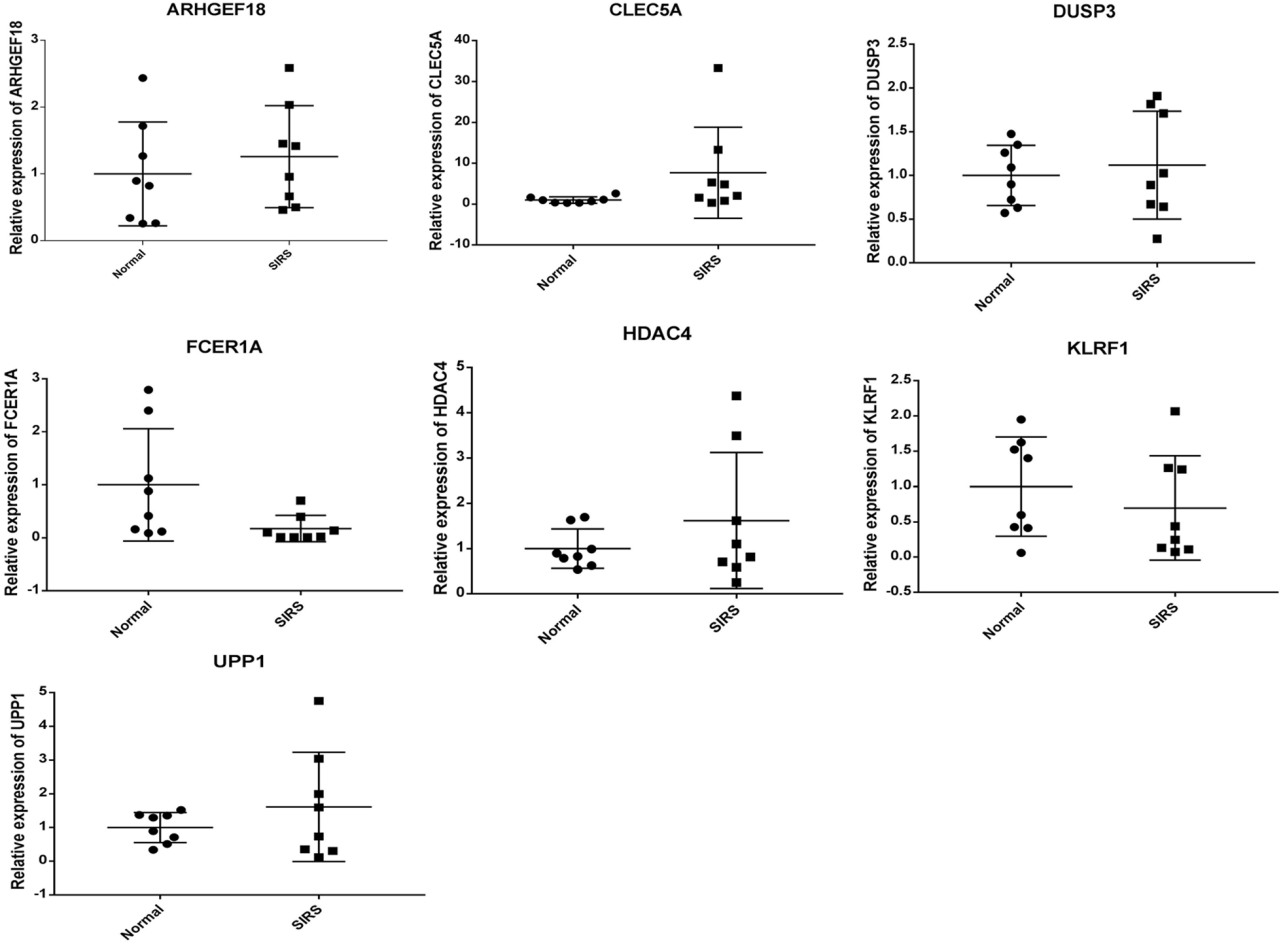
RT-PCR validation of ARHGEF18, CLEC5A, DUSP3, FCER1A, HDAC4, KLRF1 and UPP1 in blood samples. The vertical coordinate and horizontal coordinates represent relative gene expression and sample type, respectively. Normal, normal controls; SIRS, septic shock patients

The information of enrolled individuals is shown in Table 4. According to diagnostic analysis, prognostic analysis and literature reports, ARHGEF18, CLEC5A, FCER1A, HDAC4, KLRF1, DUSP3 and UPP1 were selected for RT-PCR verification. The primers are shown in Table 5. The results showed that CLEC5A, DUSP3, HDAC4 and UPP1 were up-regulated trend and FCER1A and KLRF1 were down-regulated trend (Fig. 8). The genes expression trend in the verification result was consistent with the bioinformatics analysis, except for ARHGEF18. Small sample size may cause some inconformity. In addition, further research is needed.

**Table 4:** Clinical information of patients and normal controls in the RT-PCR

S/N	Age	Sex	Family history	Body temperature (℃)	Heart rate (times/min)	Other complications
S	21	Male	No	39.3	128	Anemia
S	72	Female	Diabetes	40	117	Unconscious
S	53	Female	No	39.2	116	Stomach ache
S	50	Female	No	38.6	127	Stomach ache
S	84	Female	No	37.7	104	Expectoration
S	60	Male	No	39	94	No
S	62	Female	No	37.5	110	Abdominal pain, Lung infection
S	53	Male	No	37.8	118	Difficulty breathing
N	83	Female	No	36.5	135	No
N	22	Female	No	36.4	101	No
N	63	Female	No	37.3	122	No
N	66	Female	No	36.7	79	No
N	68	Female	No	36.8	89	No
N	45	Male	No	36.5	85	No
N	69	Male	No	36.5	95	No
N	65	Male	No	36.6	112	No

*P* septic shock patients, *N* normal control

**Table 5:** Primer sequence in the RT-PCR

Primer name	Primer sequence (5ʹ to 3ʹ)
GAPDH-F (Internal reference)	5-CTGGGCTACACTGAGCACC-3
GAPDH-R (Internal reference)	5-AAGTGGTCGTTGAGGGCAATG-3
ACTB-F (Internal reference)	5-TCCGCAAAGACCTGTACGC-3
ACTB-R (Internal reference)	5-CTGGAAGGTGGACAGCGAG-3
ARHGEF18-F	5-ACGCCAGCAAAGAAGACGT-3
ARHGEF18-R	5-CAGGCGGTCATCAGTGGTT-3
CLEC5A-F	5-GCAATTGTCAACACGCCAGA-3
CLEC5A-R	5-GCCAATGGTCGCACAGTTG-3
DUSP3-F	5-GAAGATATGGGGCAACTGGA-3
DUSP3-R	5-ATGCACGTGTTCAGCTTGAG-3
FCER1A-F	5-GTTCTTCGCTCCAGATGGC-3
FCER1A-R	5-TTGTGGAACCATTTGGTGGAA-3
HDAC4-F	5-AGCGTCCGTTGGATGTCAC-3
HDAC4-R	5-CCTTCTCGTGCCACAAGTCT-3
KLRF1-F	5-GGGAATATCTGGAACCGTGA-3
KLRF1-R	5-CTGCAGATCTCGAAGCACAA-3
UPP1-F	5-TGGAGTCCTCGGTGTTTGC-3
UPP1-R	5-GCTCAGGCCTTGCTCAGTT-3

## Discussion

Based on the machine learning method, 15 DEmRNAs, such as HMGN3, CACNA2D3, DUSP3, MGST1, CLEC5A, KIF1B, RAB13, ARHGEF18 and FCER1A, were determined as the superlative diagnostic gene biomarkers. The final survival analysis showed that only FCER1A and ARHGEF18 had obvious prognostic value.

HMGN3 (high mobility group nucleosomal binding domain 3) plays an important regulatory role in pancreatic cells [33]. In patients with sepsis, high blood sugar is a risk factor for poor prognosis. During sepsis, the rapid changes in microvascular circulation in skeletal muscle have a serious hindrance to the delivery of insulin [34]. HMGN3 can reduce the level of glucagon in the plasma [35] to maintain stable blood sugar level in the body. In this study, HMGN3 was down-regulated in patients, which laid the foundation for further verification of the role in sepsis.

MGST1 (microsomal glutathione s-transferase 1), an important redox and detoxification enzyme, play a crucial role in cell defense and hematopoiesis [36, 37]. CLEC5A (c-type lectin domain containing 5A) is a Syk (spleen tyrosine kinase) coupled c-type lectin, mainly expressed in myeloid cells, such as macrophages and neutrophils [38], participates in host defense, inflammation, platelet activation and development [39]. KIF1B (kinesin family member 1B) gene belongs to the kinesin superfamily, which is responsible for encoding proteins that transport mitochondria and synaptic vesicle precursors within the cell [40]. In addition, KIF1B is found to be a tumor suppressor gene [41, 42], which has a potential role in mitochondrial morphological changes. KIF1B and mitochondrial metalloproteinase YME1L1 (YME1 like 1 ATPase) coordinately regulate mitochondrial fission to induce mitochondrial apoptosis [43]. In the early stage of sepsis, released NO (nitric oxide) can directly block mitochondrial respiration and cause body shock when accumulated to a certain degree [6]. The potential role of KIF1B in mitochondria suggested that it may play a role in septic shock. RAB13 (RAB13, member RAS oncogene family) is present in all macrophage-related cells [44]. In our study, MGST1, CLEC5A, KIF1B and RAB13 were all up-regulated in patients. This showed that MGST1, CLEC5A, KIF1B and RAB13 could play a crucial role in septic shock.

KLRF1 (killer cell lectin like receptor F1) is an activating homodimeric C-type lectin-like receptor, which plays an important role in regulating the activity of natural killer cells and monocytes [45]. Recently, UPP1 (uridine phosphorylase 1) is reported to play an important role in immune and inflammatory biological process of disease [46–48]. Previous studies have found that the expression of UPP1 is increased in the brain of sepsis rats [49]. HDAC4 (histone deacetylase 4) plays an important regulatory role in sepsis and may be an effective target for sepsis treatment [50, 51]. The expression level of NARF (nuclear prelamin A recognition factor) in multiple sclerosis (a chronic neuroinflammatory disease) was increased [52]. So far, we have not found any studies on ECRP (ribonuclease A family member 2C, pseudogene) and LHFPL2 (LHFPL tetraspan subfamily member 2) in inflammatory or immune diseases. This article may first report that ECRP and LHFPL2 play a role in the progression of septic shock. In our study, KLRF1 (down-regulated), UPP1 (up-regulated), HDAC4 (up-regulated), NARF (up-regulated), ECRP (up-regulated) and LHFPL2 (up-regulated) were all abnormally expressed and could be considered as potential diagnostic biomarkers. These results suggested that KLRF1, UPP1, HDAC4, NARF, ECRP and LHFPL2 play a key role in septic shock. It provides a potential direction for further research on septic shock.

The protein encoded by ARHGEF18 (Rho/Rac guanine nucleotide exchange factor 18) plays an important role in activating eosinophils and other white blood cells [53]. Sepsis is a high-risk disease caused by host reaction disorder and endangering the safety of life [54]. Eosinophils are components of white blood cells of the immune defense system, and play a role in evolution of inflammation and disease [55, 56]. FCER1A (Fc fragment of IgE receptor Ia) is an IgE receptor (immunoglobulin receptor), which is the initiating factor of allergic reactions and plays a role in allergic inflammation [57, 58]. The interaction between FCER1B and other immunoglobulin-related inflammatory genes will increase the risk of asthma [59]. In this study, ARHGEF18 and FCER1A were related to survival. In the enriched GO function, ARHGEF18 is mainly involved in regulating cell death and apoptosis. FCER1A is mainly involved in regulating immune regulation and metabolic processes. This further showed that ARHGEF18 and FCER1A may be related to the survival of septic shock patients.

The MAPK (mitogen-activated protein kinase) signaling pathway play a crucial part in the regulation of diseases, such as anti-inflammatory, analgesic, protective injury, etc. [60]. MAPK contains three sub-pathways p38MAPK (p38 mitogen-activated protein kinase), ERK-1/2 (extracellular signal-regulated kinase), and JNK (c-Jun-terminal kinase) [61, 62]. Among them, the p38MAPK and JNK signaling pathways play a role in hamowanie wzrostu, inflammation and pro-apoptotic signaling [60]. MAPK pathway can be activated by extracellular signals, such as cytokines involved in inflammatory response, growth factors that regulate growth and metabolism, bacterial complexes [60]. Inhibiting the activation of the MAPK pathway can reduce lung injury caused by septic shock [63]. In the KEGG enrichment, CACNA2D3 and DUSP3 were taken part in the MAPK signaling pathway. CACNA2D3 (calcium voltage-gated channel auxiliary subunit alpha2delta3) plays an important role in canceration [64–66]. CACNA2D3 is expressed in low levels in endometrial cancer tissues and cells [64]. Overexpression of CACNA2D3 in vitro significantly inhibits tumor cell proliferation and migration [64]. CACNA2D3, as a new tumor suppressor gene, can significantly inhibit lymph node metastasis of esophageal squamous cell carcinoma in clinical studies [67]. Lymph nodes are immune sites for lymphocytes, which lays the foundation for studying the role of CACNA2D3 in septic shock. DUSP3 (dual specificity phosphatase 3), also called VHR (vaccinia-H1 related phosphatase), is a founding member of the bispecific protein phosphatase group [68]. DUSP3 plays a role in Staphylococcus aureus infection [69], DUSP3, a positive regulator of innate immune response [70], is the main protein tyrosine phosphatase in macrophages mediating cellular processes (including immune responses) [71]. This further illustrates that MAPK signaling pathway may play an irreplaceable role in septic shock by regulating related genes such as CACNA2D3 and DUSP3.

However, this study has certain limitations. Firstly, the sample size of the RT-PCR experiment is small, which may lead to a certain degree of error. More blood samples from septic shock patients are further needed to verify the expression of the identified mRNAs. Secondly, the molecular mechanism of DEmRNAs during septic shock has not been studied. More experiments are needed to further research the underlying mechanism of the disease.

## Conclusions

In this study, in order to identify potential diagnostic gene biomarkers of septic shock, machine learning method was performed, followed by prognostic analysis. 15 superlative diagnostic gene biomarkers (KLRF1, UPP1, RAB13, KIF1B, CLEC5A, NARF, DUSP3, FCER1A, CACNA2D3, HMGN3, ECRP, HDAC4, LHFPL2, MGST1 and ARHGEF18) for septic shock were identified by machine learning analysis. It is worth mentioning that ARHGEF18 and FCER1A were related to survival. CACNA2D3 and DUSP3 participated in MAPK signaling pathway to regulate septic shock. Identified diagnostic gene biomarkers may be helpful in the diagnosis and therapy of patients with septic shock. This study can provide a basis for the research of septic shock.

## Supplementary Information

**Additional file 1: Figure S1.** Batch effect processing between different data sets.

**Additional file 2: Figure S2.** Validation analysis in GSE95233 data set. A: Electronic expression validation of 15 diagnostic gene biomarkers in GSE95233 data set. **** represent P < 0.0001; B: ROC curve of DT classifier; C: ROC curve of RF classifier; D: ROC curve of SVM classifier. AUC: area under curve, ROC: receiver operating characteristic. 

**Additional file 3: Table S1.** All DEmRNAs.

**Additional file 4: Table S2.** Calculation of Matthew’s Correlation Coefficient.

## Data Availability

All data generated or analyzed during this study are included in this published article. The data sets (GSE4607, GSE13904, GSE26378, GSE26440, GSE65682 and GSE95233) analysed during the current study are available in the GEO (Gene Expression Omnibus) database, persistent accessible web link to database is https://www.ncbi.nlm.nih.gov/geo/.
