# Supplementary figures and images for: Revealing potential diagnostic gene biomarkers of septic shock based on machine learning analysis
Source: BMC Infect Dis. 2022 Jan 19;22:65. doi: 10.1186/s12879-022-07056-4 (PMC8772133; doi:10.1186/s12879-022-07056-4)

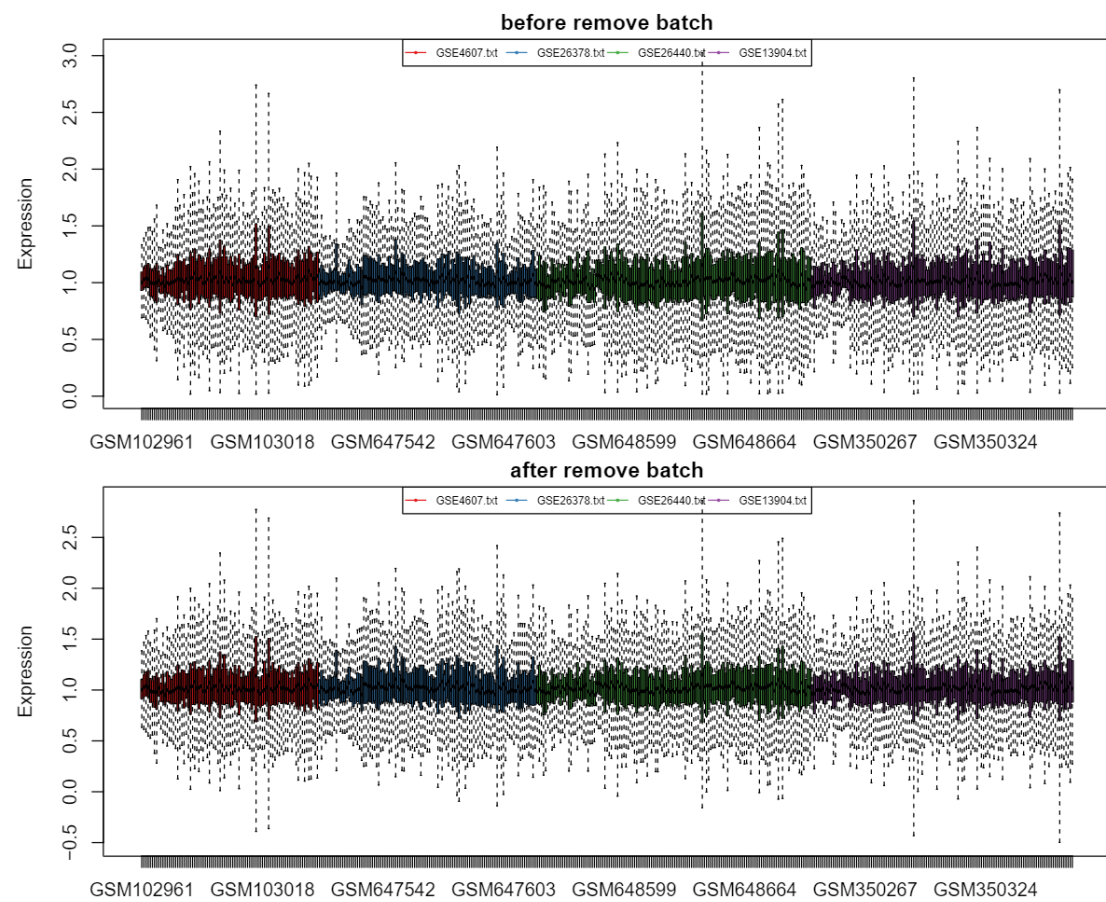

**Figure S1 Batch effect processing between different data sets.**

Supplement: Supplementary file 1 — Additional file 1: Figure S1. Batch effect processing between different data sets. [file 12879_2022_7056_MOESM1_ESM.pdf]
