# Supplementary material for: Revealing potential diagnostic gene biomarkers of septic shock based on machine learning analysis
Source: BMC Infect Dis. 2022 Jan 19;22:65. doi: 10.1186/s12879-022-07056-4 (PMC8772133; doi:10.1186/s12879-022-07056-4)
Supplement: Supplementary file 2 — Additional file 2: Figure S2. Validation analysis in GSE95233 data set. A: Electronic expression validation of 15 diagnostic gene biomarkers in GSE95233 data set. **** represent P < 0.0001; B: ROC curve of DT classifier; C: ROC curve of RF classifier; D: ROC curve of SVM classifier. AUC: area under curve, ROC: receiver operating characteristic. [file 12879_2022_7056_MOESM2_ESM.pdf]

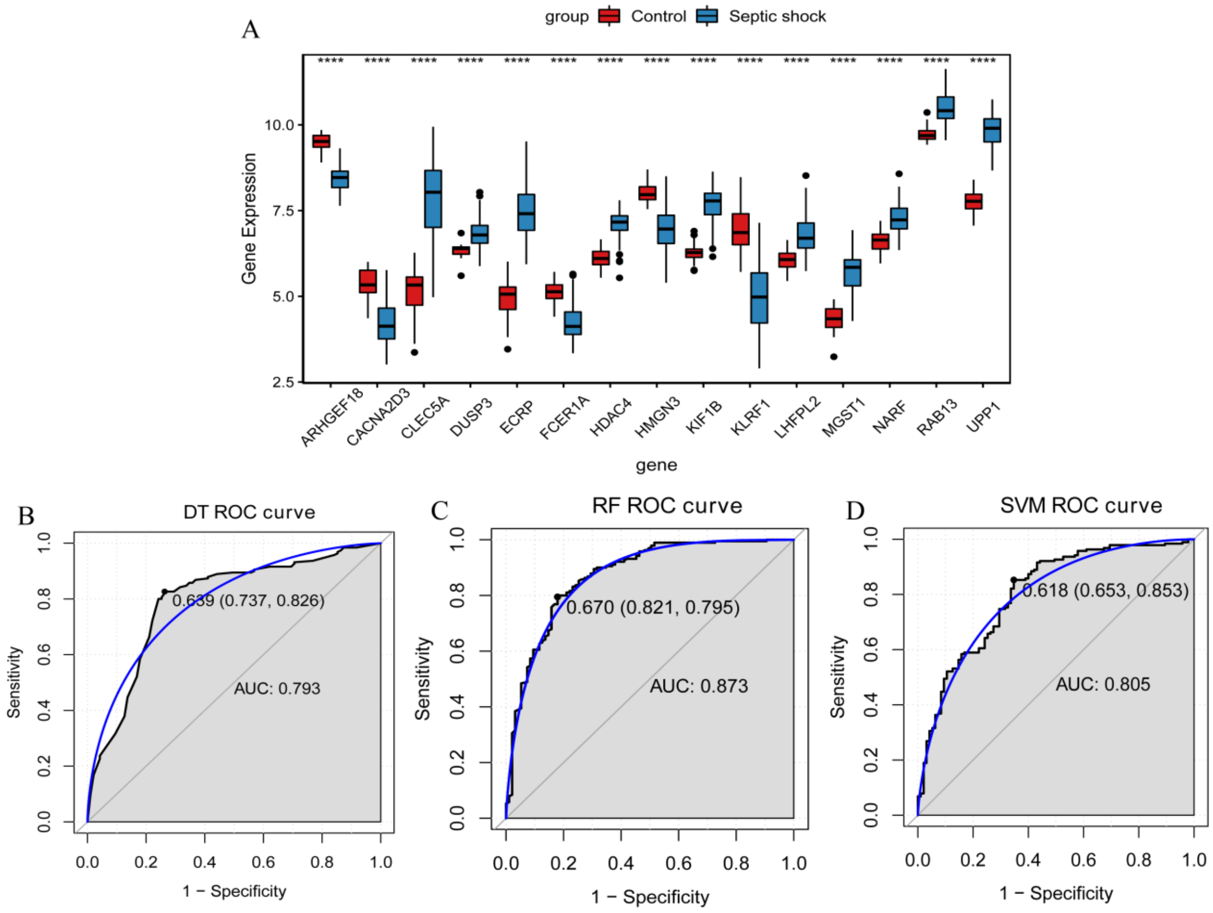

Figure S2 Validation analysis in GSE95233 data set.

A: Electronic expression validation of 15 diagnostic gene biomarkers in AA data set. **** represent $P < 0.0001$; B: ROC curve of DT classifier; C: ROC curve of RF classifier; D: ROC curve of SVM classifier. AUC: area under curve, ROC: receiver operating characteristic.
