# Supplementary material for: Revealing potential diagnostic gene biomarkers of septic shock based on machine learning analysis
Source: BMC Infect Dis. 2022 Jan 19;22:65. doi: 10.1186/s12879-022-07056-4 (PMC8772133; doi:10.1186/s12879-022-07056-4)
Supplement: Supplementary file 4 — Additional file 4: Table S2. Calculation of Matthew’s Correlation Coefficient. [file 12879_2022_7056_MOESM4_ESM.docx]

Table S2 Calculation of Matthew’s Correlation Coefficient

| Classifier | test set | validation set |
| --- | --- | --- |
| SVM | 0.964 | 0.653 |
| RF | 0.986 | 0.713 |
| DT | 0.875 | 0.647 |

DT, decision tree; RF, random forest; SVM, support vector machines.
